# Millimeter Wave Radiations Affect Membrane Hydration in Phosphatidylcholine Vesicles 

**DOI:** 10.3390/ma6072701

**Published:** 2013-07-09

**Authors:** Amerigo Beneduci, Katia Cosentino, Giuseppe Chidichimo

**Affiliations:** 1 Department of Chemistry, University of Calabria, Via P. Bucci-Cubo 17/D, Arcavacata di Rende (CS) 87040, Italy; E-Mails: katia.cosentino@unical.it (K.C.); giuseppe.chidichimo@unical.it (G.C.); 2 Adhesion and Inflammation, CNRS UMR 7333, INSERM U1067, Aix Marseille University, Luminy, Marseille 13009, France

**Keywords:** bio-mimetic membranes, phosphatidylcholine, millimeter wave exposure, deuterium quadrupole splitting, nuclear magnetic resonance

## Abstract

A clear understanding of the response of biological systems to millimeter waves exposure is of increasing interest for the scientific community due to the recent convincing use of these radiations in the ultrafast wireless communications. Here we report a deuterium nuclear magnetic resonance spectroscopy (^2^H-NMR) investigation on the effects of millimeter waves in the 53–78 GHz range on phosphocholine bio-mimetic membranes. Millimeter waves significantly affect the polar interface of the membrane causing a decrease of the heavy water quadrupole splitting. This effect is as important as inducing the transition from the fluid to the gel phase when the membrane exposure occurs in the neighborhood of the transition point. On the molecular level, the above effect can be well explained by membrane dehydration induced by the radiation.

## 1. Introduction

The increasing interest in the biological effects of millimeter waves (MMWs; 30–300 GHz), expressed in recent years, is mainly due to the significant development of millimeter wave sources for ultrafast wireless communication systems [1]. Despite the low power density emitted from these sources (below 1 mW/cm^2^), exposure to this radiation could be potentially dangerous for human beings [2,3,4,5]. Even though no significant thermal effects have been detected, several non-thermal biological effects have been claimed [2,3,5]. Reversible morphological and ultrastructural alterations were observed on different human tumor cell systems after long term exposure to radiation in the 53–78 GHz frequency range [6,7,8]. These effects were generally associated with a significant inhibition of cell proliferation [6,7,8]. In addition, it has been shown that these radiations might affect the kinetics of glucose metabolism in leukemia cells, enhancing the glycolytic aerobic pathway. This indicates that the exposed system needs to produce extra-bioenergy as a defense/reparatory response to the radiations, which results in a significant decrease in the proliferation rate without significant cell death [9]. Furthermore, it was also shown that these radiations can induce a reversible externalization of phosphatidylserine molecules in cells exposed* in vitro*, thereby affecting the structural state of phospholipids in biomembranes [10]. In contrast to these results, exposure performed at 42 GHz and 60 GHz did not result in any significant genotoxic effect or evidence of cellular stress [11,12,13]. For a detailed overview on the topic we refer readers to the recent review by Zhadobov* et al.* [5].

Strong evidence shows that biological membranes are likely to be the main target of millimeter waves with frequency in the range 1–80 GHz [4]. In recent years, model membranes such as phospholipid monolayers [14], multilamellar [15] and unilamellar [16,17] phospholipid vesicles, have been increasingly used to investigate the effect of millimeter waves on the membrane structure and functions. Effects on the permeability of cationic liposomes were observed under exposure at 130 GHz [16]. More recently, it has been shown that millimeter wave exposure in the frequency range 53–78 GHz affects the permeability properties of giant unilamellar vesicles (GUVs) to water under osmotic stress conditions and the kinetics of the aging processes in large unilamellar vesicles (LUVs) [17]. Both these effects have been explained in terms of a partial dehydration and consequent increased rigidity of the lipid membrane [17]. In addition, physical changes in GUVs, such as elongation, induced diffusion of fluorescent dye di-8-ANEPPS into the bilayer and increased attraction between vesicles, were observed under radiation exposure at 53.37 GHz [18]. The action of the field on charged and dipolar residues, located at the membrane-water interface, is thought to be the major factor determining the overall perturbation of these vesicles [17,18]. This hypothesis is reinforced by ^2^H-NMR molecular spectroscopy analysis of the water bound to the membrane in multilamellar lipid vesicles exposed at 53–78 GHz, where an upward shift of the fluid-to-gel lipid transition temperature was observed [15].

**Figure 1:**
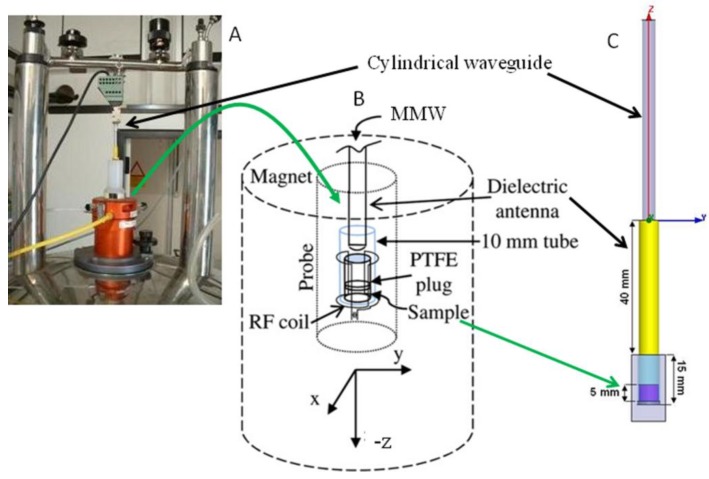
Exposure set-up showing the millimeter-waves applicator connected to a diamagnetic cylindrical waveguide inserted into to the nuclear magnetic resonance (NMR) magnet (**A**); The waveguide terminates with a dielectric Teflon antenna located in the NMR probe over the sample (**B**). Model of the scenario for the dosimetric assessment: the millimeter waves (MMWs) applicator inserted into a cuvette with the exposed sample.

Here we provide some results on the response of deuterium labeled 1,2-dimyristoyl-sn-glycero-3-phosphatidylcholine/^2^H_2_O (DMPC/^2^H_2_O) membranes to millimeter waves (53–78 GHz) at the physiologic temperature (37 °C), obtained by ^2^H-NMR spectroscopy. Since the heavy water quadrupole splitting, ∆ν_q_, can be related to changes in the bilayer structures [15,19,20], it is a useful probe of membrane surface geometry and membrane hydration. For a powder sample of multilamellar vesicles (MLVs), ∆ν_q_ is the distance (Hz) between the highest peaks of the powder spectrum (see Figure 1A) and is given by the sum of the contribution of water bound to different sites along the phosphocholine headgroup of the membrane [15,19,20]:

(1) $$∆\nu_{q}=\frac{3}{4}\nu_{q}\sum p_{b,i}S_{b,i}$$

where, *ν_q_* = 220 KHz is the water quadrupole coupling constant; *p_b,i_* and *S_b,i_* are, respectively, the fraction and the molecular order parameter of bound water at the *i*th binding site. The last parameter takes the fast anisotropic molecular reorientations of water molecules at the membrane interface into account.

## 2. Materials and Methods

### 2.1. Sample Preparation

DMPC was purchased from Sigma Aldrich with a 98.8% purity and used without further purification. ^2^H_2_O was obtained from Cambridge Isotope Inc. having a purity of 99.98%. DMPC phospholipids labeled with deuterium on the C2 carbon atoms of the sn1 and sn2 chains (DMPC-d4) were synthesized as described elsewhere [21] and purified by column chromatography. Their purity was further checked by thin layer chromatography and used without further purification. Mixtures of pure DMPC with deuterium labeled DMPC were prepared by solubilization in chloroform. The solvent was removed by blowing a gentle stream of nitrogen over the sample and then placing it under high vacuum overnight. A white dry fluffy powder was thus obtained. DMPC powders were dried to a constant weight in vacuum at room temperature. Multilamellar vesicles (MLVs) were prepared by adding an aliquot of water (either H_2_O or D_2_O) to a known amount of dry phospholipids in a 5 mm quartz tube that was hermetically sealed. Samples were weighted and stored at 37 °C for three days. Before performing the exposure treatment, ^2^H-NMR spectra were recorded over 48 h to ensure the time stability of the observed splitting. After any exposure, samples were weighted to check out for water evaporation.

### 2.2. ^2^H-NMR Acquisitions

DMPC/D_2_O and DMPC-d4/H_2_O phase transition were monitored with a precision of 0.2 °C, by a Bruker VT 2000 unit (Bruker Biospin, Germany). Samples were brought into the fluid phase, above T_m_, and their temperature was gradually decreased in steps of 0.2 °C in order to enter the gel phase. For each new T value, the system was equilibrated for at least 30 min prior to acquisition. The reverse process (gel-to-fluid phase) was studied by increasing the temperature in the same way. For DMPC-d4 samples, spectra were acquired using a spectral width of 100 KHz and accumulating 3000–7000 FIDs. Spectra were de-Paked using the dmfit free program [22] according to the procedures described by the authors. FIDs were apodized with an exponential decay corresponding to a line broadening of 200 Hz, prior to perform the FT.

Segmental order parameters of the acyl chains of the phospholipids were calculated by the quadrupole splitting of the deuterium nuclei in the C–^2^H bond, expressed as $\left|∆\nu_{Q}\right|=\frac{3}{2}\nu_{Q}\left|S_{2}^{CD}\right|\left|\frac{3cos^{2}\vartheta -1}{2}\right|$, where $\nu_{Q}$ is the quadrupole coupling constant equal to 167 KHz and ϑ the angle between the director of the lamellar phase and the static magnetic field. $∆\nu_{Q}$ was measured as the distance of the two inner singularities of the powder spectrum (ϑ = 90°) [23,24,25,26].

### 2.3. Real-Time NMR Acquisition under Exposure

MMW exposure was performed by means of a microwave wide-band generator (Amphit-32, MicroMedTech, Nizhny Novgorod, Russia) in the 53.57–78.33 GHz frequency (f) range and incident power densities (IPD) of 0.0035–0.010 mW/cm^2^. Further experiments were carried out irradiating the samples with monochromatic millimeter sources at 53.37 GHz, 62.10 GHz and 65.00 GHz (IMG series, MicroMedTech, Nizhny Novgorod, Russia).

To allow real time detection of NMR spectra during exposure, the MMW generator was connected to a diamagnetic cylindrical waveguide of variable length terminating with a dielectric Teflon antenna inserted into the NMR probe with a vertical coil (Figure 1). Samples were subjected either to MMW radiation or to sham exposure, which corresponds to the same set-up but with the generator turned off (Incident power density = 0). NMR spectra were acquired in real-time in strictly controlled temperature conditions. 

## 3. Results and Discussion

### 3.1. Exposure at 53–78 GHz Causes a Time-Dependent Decrease of the Quadrupole Splitting

**Figure 2:**
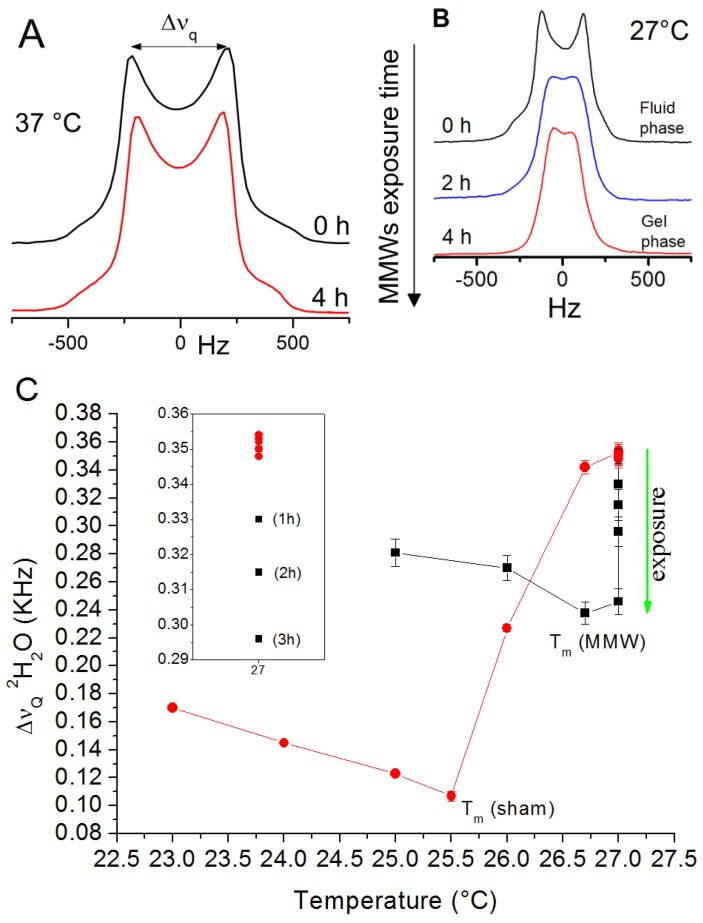
Effects of millimeter waves (53–78 GHz) on deuterium labeled 1,2-dimyristoyl-sn-glycero-3-phosphatidylcholine (DMPC) vesicles measured as real-time changes of the ^2^H-NMR line shapes at 37 °C (**A**) and 27 °C (**B**) during exposure. The heavy water quadrupole splitting (∆ν_q_) is defined as the distance (in Hz) between the peaks of the spectrum. Quadrupole splitting behavior of DMPC/^2^H_2_O multilamellar vesicles (MLVs) (*n*_w_ = 11) under sham (●) and MMWs exposure (■) in the transition region (T_m_ = phase transition temperature).

^2^H-NMR spectra of the DMPC/^2^H_2_O membrane acquired at 37 °C before and after 4 h of millimeter wave wide-band exposure in the range 53–78 GHz are compared in Figure 2A. The radiation induced a decrease of the water quadrupole splitting of about 25% (Table 1). This effect could be observed only after some exposure time (up to several hours). 

Spectra acquired at 27 °C under the same exposure conditions, showed a similar trend (Figure 2B) [15]. Notably, in this case, the effect of the quadrupole splitting decrease is large enough to induce the fluid-to-gel phase transition at 27 °C (Figure 2C), where in the sham exposed (control) system the main phase transition occurs at 25.5 °C. 

It is worth noting that the decrease of the water quadrupole splitting is reversible and the membrane system relaxes back to its pre-exposure state after a few hours from the end of the exposure. The time-dependent nature of this phenomenon suggests the hypothesis that the energy provided by the radiation is accumulated by the system over time until it becomes sufficient to induce the observed effects.

This idea, first conjectured by Fröhlich [27], has recently been revised by Reimers* et al.* [28], but still needs to be definitely proved.

### 3.2. The Observed Effects Have a Non-Thermal Nature

**Figure 3:**
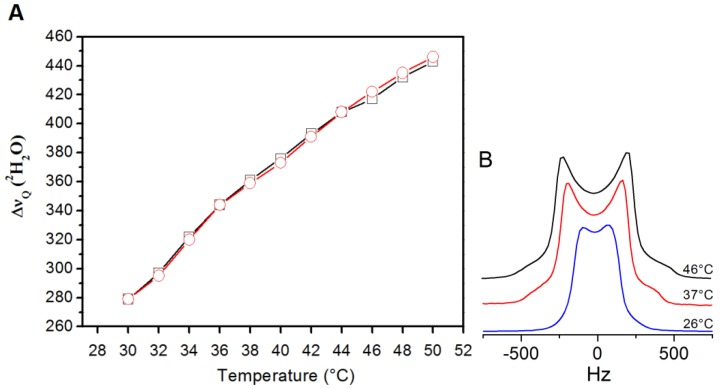
Temperature effect on the heavy water quadrupole splitting (∆ν_q_) of DMPC vesicles with a water/lipid mole ratio *n* = 12 in the range 30–50 °C. (**A**) Heating (○) and cooling (□) curves and (**B**) corresponding ^2^H-NMR lineshape changes for selected temperatures.

To prove the nonthermal nature of the effects observed under exposure, we have followed the change in the quadrupolar splitting by increasing (and decreasing) the temperature in the range 30–50 °C (Figure 3A). A temperature increase induces an increase in the quadrupolar splitting (Figure 3B). Therefore the decrease in the quadrupolar splitting observed under exposure (Figure 2) cannot be due to heating effects. Further, accurate dosimetric analysis (see supplementary information) confirms that only negligible thermal effects were induced on the membrane during irradiation. Indeed, in the exposure conditions described, an average whole sample average specific absorption rate (SAR) ranging from 1 to 3 mW/kg was calculated, depending on the frequency and on the sample thickness, with average time rate of initial temperature rise of the order of 10^−6^–10^−7^ K/s. Furthermore, the largest estimated thermal gradient (74 GHz, 20 μW output power) was ~0.08 < 0.1 °C (see supplementary materials and [15]).

### 3.3. Dependence on the Water/Lipid Mole Ratio

**Figure 4:**
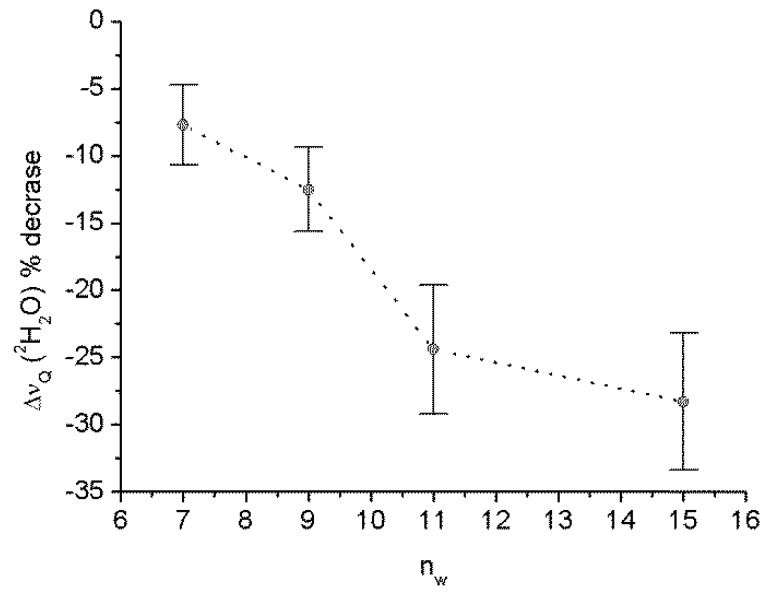
Heavy water quadrupole splitting reduction induced by 4 h MMWs exposure in the wide-band mode on DMPC/^2^H_2_O MLVs samples with different water/mole lipid ratio (*n*_w_). Points and error bars are, respectively, means and standard deviations relative to three independent experiments.

Figure 4 shows the heavy water quadrupole splitting reduction (%) induced by 4 h millimeter wave exposure as a function of the membrane hydration regime,* i.e.*, the water/lipid mole ratio (*n*_w_). It can be seen that the effect of the radiation increases with the increase in *n*_w_, suggesting that this effect is related to some change in the membrane hydration.

### 3.4. Frequency-Dependent Effects

In order to probe whether the above effects were frequency-specific in the range of frequencies used, we exposed the sample to three different frequency points: 53.37 GHz, 62.1 GHz and 65.00 GHz. 

**Table 1:** Effects induced by extremely high frequency millimeter waves on DMPC multilamellar vesicles (*n*_w_ = 11) in the fluid phase at 37 °C probed by ^2^H-NMR spectroscopy.

**Exposure frequency mode**	**Exposure conditions**		**Effects**
	Frequency (GHz)	(SAR) ^a^ mW/kg	∆ν_q_ Decrease (%) ^b^
wide-band	53–78	1–3	25 ± 5%
frequency point	53.37	2.7	nd ^c^
	62.10	1.2	22 ± 5
	65.00	1.0	nd ^c^

^a^ As calculated in [12]; ^b^ Values averaged over three independent experiments; ^c^ Not detected.

Table 1 summarizes the changes detected in the heavy water NMR powder spectra on DMPC multilamellar vesicles by the different exposure conditions tested, after 4 h of continuous wave exposure. Among the three single-mode frequencies, only the exposure at 62 GHz induced a change of the quadrupole splitting similar to the one observed under wide-band mode exposure conditions. This preliminary result suggests a specific frequency response of the investigated membrane model system in the 53–78 GHz range.

### 3.5. Millimeter Waves Change the Bound Water Partitioning at the Membrane Polar Interface Increasing the Membrane Rigidity

The observed water quadrupole splitting decrease can be interpreted as a change of the water distribution among the various binding sites of the phosphocholine headgroup, *i.e.*, as a change of *p_b,i_* (Equation (1)). 

The preliminary data reported in Table 2 on DMPC membrane labeled with deuterium in the acyl chains, show that this water distribution rearrangement at the headgroups is coupled to a significant change of the C1–C2 segment orientation of the sn2 chain. Table 2 reports the S_2_^CD^ segmental order parameters for the DMPC-d4/H_2_O bio-membrane. Under exposure, we have observed a significant increase of the absolute value of the intramolecular CD bond order parameter relative to the deuteron pro-R in position C2 of the sn2 phospholipid chain (Table 2). It is worth noting that the change induced on this parameter increases with increasing *n*_w _(Table 2), thus supporting the results of Figure 4. The sn2 pro-R parameter is very sensitive to structural changes of the headgroups conformation (especially near the phase transition) and its increase suggests that the beginning of the sn2 chain tends to be more extended into the bilayer core, reflecting an increase in the chain packing [29]. 

On the other hand, this interpretation is consistent with the increase of the phospholipid lateral pressure in phosphocholine monolayers exposed at 61 GHz and at a few pW/cm^2^ [14] as well as to a decrease of the membrane permeability to water as observed in GUVs under osmotic stress [17]. 

**Table 2:** S_2_^CD^ order parameters for the position C2 of the sn1 and sn2 lipid chains for two different hydration regime (*n*_w_)

Order parameter	DMPC/H_2_O			
S_2_^CD^	*n*_w_ = 11		*n*_w_ = 17	
chain segment	Sham	Exposed	Sham	Exposed
sn1	−0.210	−0.211	−0.198	−0.197
sn2 (pro-R)	−0.156(0.001)	−0.162(0.003)	−0.130(0.002)	−0.136(0.002)
sn2 (pro-S)	0.112	0.110	0.090	0.090

Note: sn2 (pro-R) values are expressed as mean (sd) of three independent experiments.

According to the literature, the two deuterons on position C2 of the sn1 chain are magnetically equivalents and they exhibit unique order parameter with negative sign due to geometrical reasons [29]. In fact, in the all-trans chain conformation, typical of the fluid phase, the C–D bonds form approximately tetrahedral angle with respect to the normal to the bilayer, having the beginning of the sn1 chain an extended average conformation. Conformational disorder is induced by increasing the temperature, as it is shown by the markedly decrease of the sn1-S_2_^CD^, showing that the sn1-S_2_^CD^ is very sensitive to thermal variations [29]. In the sn2 chain, the two deuterons on the alpha carbon atom are magnetically nonequivalent and two different order parameters are observed: one positive, assigned to the pro-S deuteron and the other one negative, associated to the pro-R deuteron. The non-equivalence of the deuterons has been used to determine the average orientation of the 0=C–C group linked to the glycerol backbone [29]. It has been shown that, due to allowed trans-gauche isomerization of the C1–C2 bond, this segment of the sn2 chain adopts a favorable, thermally insensitive bent conformation with respect to the normal to the bilayer [29]. In particular, the pro-S S_2_^CD^ is not sensitive to either thermal variations or structural changes [29]. On the other hand, the pro-R S_2_^CD^ is poorly affected by thermal variation (up to 40 °C) while it is very sensitive to structural changes that affect the geometrical orientation of the C1–C2 segment, which is the case when the system approaches the phase transition [29]. This parameter can be therefore used as a peculiar sensor for the reorientation of the beginning of the sn2 chain. From Table 2 it can be seen that radiation exposure significantly increases the absolute value of the pro-R S_2_^CD^ order parameter, after about 4 h of exposure. Interestingly, it does not affect either the pro-S S_2_^CD^ or the sn1-S_2_^CD^ parameters. In our exposure experiment, no variation of temperature and composition of the sample were allowed, thus the pro-S S_2_^CD^ and the sn1-S_2_^CD^ do not change significantly. On the other hand, a larger value of the $\left|\text{pro}-{\text{R S}}_{2}^{\text{CD}}\right|$ means that the C–D bond undergoes restricted dynamics of the trans-gauche conformational isomerization around the C1–C2 bond. This is related to a change in the orientation of the C1–C2 segment of the sn2 chain that adopts a less bent orientation [29]. Therefore, in the exposed bio-membranes, the sn2 chain tends to be more extended along the normal to the bilayer. We then surmise that the structural changes of the headgroups conformation related to the radiation induced changes in the water partitioning at the membrane interface, are coupled to an increase of the rigidity of the bilayer.

## 4. Conclusions

The above results can be summarized as follows:

(1) The time-dependent nature of the effect suggests that accumulation phenomena occur,* i.e.*, that the electromagnetic energy is mainly stored in the system during the exposure as chemical potential and is not simply thermalized.
(2) The effect is amplified in proximity to the membrane phase transition point. It suggests that cooperative phenomena occur, due to long range interaction among the membrane subunits.
(3) The MMWs-induced water quadrupole splitting reduction suggests a change in the water partitioning at the membrane interface.
(4) The strong dependence of the exposure effects on the water/lipid mole ratio reveals the critical role of water in mediating the MMWs/biomembrane interaction: as the water/lipid mole ratio increases, increasing mm-wave energy is deposited into the sample, since water strongly absorbs the radiation, leading to a greater effect.

Altogether, these effects indicate that extensive exposure to millimeter waves (53–78 GHz) causes molecular changes at the water-bilayer interface. These changes can have far reaching consequences on the properties and function of biological membranes. Lateral diffusion of lipids and proteins inside the membrane, passive diffusion of small molecules through the bilayer, conformational changes of proteins inside the membrane, are all processes which are strictly dependent on the membrane hydration and permeability, and any change in the membrane properties can affect regulations and functions of biological cells.

## Supplementary Materials

Supplementary File 1
