# Supplementary material for: Millimeter Wave Radiations Affect Membrane Hydration in Phosphatidylcholine Vesicles
Source: Materials (Basel). 2013 Jul 9;6(7):2701–12. doi: 10.3390/ma6072701 (PMC5521226; doi:10.3390/ma6072701)
Supplement: Supplementary File 1 [file materials-06-02701-s001.pdf]

*Materials* **2013**, *6*, 2701-2712; doi:10.3390/ma6072701

OPEN ACCESS

**materials**

**ISSN 1996-1944**

www.mdpi.com/journal/materials

*Article*

# Supplementary Information

## 1. Dosimetric Assessment

At these frequencies, the penetration depth of the radiation is rather small (~1.2 mm) and a non uniform electric field distribution in the membrane sample is observed (Supplementary Figure S1), leading to thermal gradients within the sample. Unfortunately, we could not perform temperature measurements. However, we estimated the largest thermal gradient between the local maximum and minimum peak SAR to be <0.1 ℃ after 4 h of exposure. This value was determined by integrating equation 1 over the whole exposure time in the worst case scenario, *i.e.*, assuming that all heat transfer mechanisms (conduction, convection, *etc.*) could be neglected except millimeter radiation energy deposition. It is worth to highlight that heat transfer convection mechanisms should be disabled here due to the rather viscous nature of the membrane samples used [1].

$$SAR = c\left.\frac{dT}{dt}\right|_{t=0} \quad (1)$$

where, $\frac{dT}{dt}$ is the time rate of initial temperature rises, proportional to the SAR in the first few seconds of exposure over which heat diffusion and convection do not occur [2,3]; $dT$ is the temperature increment, $dt$ the duration (s) over which $T$ is measured and $c$ is the specific heat, assumed to be that of water $c$ = 4186 J/kg K.

**Figure S1.** E-field distribution on the XY (**A**) and YZ (**B**) planes (65 GHz; 1 W incident power).

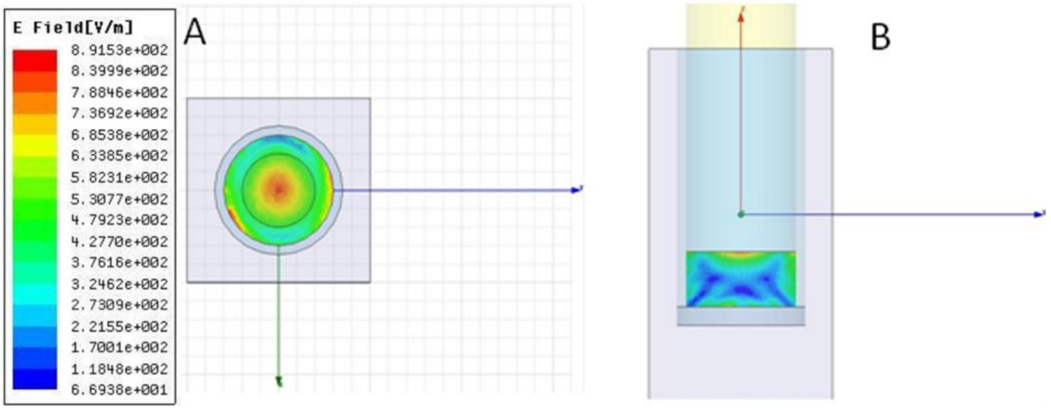

## References

1. Khizhnyak, E.P.; Ziskin, M.C. Temperature Oscillations in Liquid Media Caused by Continuous (Nonmodulated) Millimeter Wavelength Electromagnetic Irradiation. *Bioelectromagnetics* **1996**, *17*, 223–229.
2. Calabrese, M.L.; D'Ambrosio, G.; Massa, R.; Petraglia, G.A. High-efficiency waveguide applicator for *in vitro* exposure of mammalian cells at 1.95 GHz. *IEEE Trans. Millim. Theory Tech.* **2006**, *54*, 2256–2264.
3. Kühn, S.; Kuster, N. Experimental EMF exposure assessment. In *Handbook of Biological Effects of Electromagnetic Fields: Bioengineering and Biophysical Aspects of Electromagnetic Fields*, 3rd ed.; Barnes, F.S., Greenebaum, B., Eds.; Taylor and Francis (CRC Press): Boca Raton, FL, USA, 2007; pp. 381–409.

© 2013 by the authors; licensee MDPI, Basel, Switzerland. This article is an open access article distributed under the terms and conditions of the Creative Commons Attribution license (http://creativecommons.org/licenses/by/3.0/).
